# What happens in vagus

**DOI:** 10.3389/fneur.2026.1901477

**Published:** 2026-10-01

**Authors:** Steven C. Cramer

**Affiliations:** Department of Neurology, University of California, Los Angeles and California Rehabilitation Institute, Los Angeles, CA, United States

**Keywords:** depression, parasympathetic, seizure, stroke recovery, vagus nerve, vagus nerve stimulation

## Abstract

The parasympathetic nervous system is phylogenetically ancient. In humans, the vagus nerve is the brain’s only pathway for bidirectional parasympathetic signaling with the thorax, abdomen, and upper pelvis. Unsurprisingly, the vagus nerve has played a significant role in scientific discovery and therapeutic applications, attracting the attention of Galen, Darwin, and Pavlov, among others. For example, Otto Loewi used the vagus nerve to gather data that confirmed Santiago Ramón y Cajal’s Neuron Doctrine. This intensive focus has led to a number of clinical advances related to vagus nerve function, including theories about mind–body connections. Interestingly, reducing vagus nerve activity has been a successful strategy for some clinical conditions, such as peptic ulcer disease and obesity. In other conditions, increasing vagus nerve activity has been successful at improving patient outcomes, for example, in refractory seizures, drug-resistant depression, and stroke recovery; in each of these three conditions, the FDA has approved vagus nerve stimulation as a therapeutic intervention. Potential future developments in the therapeutic modulation of vagus nerve activity are discussed, including its role in perinatal brain development; manipulation of the vagus nerve as a portal for CNS access, as suggested in Parkinson’s disease; and closed-loop systems that modulate vagus nerve activity in response to measurement of various forms of signaling, an approach that would be aided by the capacity to directly measure activity in individual vagus nerves.

## Introduction

Our team was paged to the emergency department (ED) to evaluate a man who had been found pulseless and apneic at home. Hours earlier, he had celebrated his 80^th^ birthday at a party with some friends. The paramedics revived him and brought him in, where an examination showed that he was in a coma. A subsequent MRI showed extensive early anoxic brain injury, and he died shortly thereafter. He had been in relatively good health, apart from osteoarthritis, gastric reflux, and well-controlled hypertension. His wife asked: “This morning he looked fine—what happened?”

When considering the cause, common conditions must be considered first, of course, such as occult coronary artery disease or a pulmonary embolism. However, the timing of his death was quite a coincidence. This raised the question: could such an anniversary have been a contributing factor?

A theory known as “The Birthday Effect” posits that a person is more likely to die on their birthday (or a similarly high-valence anniversary) compared to other days of the year (1). Notable examples include Ingrid Bergman and Betty Friedan. Famously, John Adams and Thomas Jefferson both died on 4 July 1826, the 50th anniversary of the signing of the Declaration of Independence. Data from multiple large-scale studies support this theory, although it is not without controversy. Suggested mechanisms include higher alcohol consumption, suicide, or the mind “letting go” physiologically once a key milestone is reached. In this regard, it is theorized that increased parasympathetic signaling may be a factor in deaths that occur on high-valence anniversary dates. However, this theory is based on observational data, and the full mechanisms underlying these events remain uncertain. As such, this patient’s demise may have been intertwined with altered activity in the vagus nerve, the sole conduit that the brain has for bidirectional parasympathetic signaling with the thorax, abdomen, and upper pelvis.

## Welcome to vagus

The parasympathetic nervous system is phylogenetically ancient, first appearing hundreds of millions of years ago. It has remained evolutionarily conserved since and is found in today’s reptiles, fish, and mammals. This is sensible—an organism that rests and procreates at the appropriate times had a survival advantage. In humans, parasympathetic signals generated in the body are relayed to the nucleus tractus solitarius in the medulla, which relays these signals rostrally, resulting in rapid and widespread increases in brain levels of modulatory neurotransmitters such as norepinephrine and acetylcholine.

The vagus nerve plays a major role in health and disease and has also had a pivotal role in human history and scientific discovery. It was first described in the second century by Galen. In humans, each of the two vagus nerves contains 100,000 fibers (the same number of fibers as in an Airbus A380 jet). The vagus nerve is central to a wide range of essential functions, including swallowing, sweating, salivating, and sex. It also mediates relaxation, which was anticipated by Charles Darwin in 1872 when he suggested that our emotions result from the flow of information between the heart and the brain via the vagus nerve (then called the pneumogastric nerve) (2).

The vagus nerve is also a key player in an individual’s development. It forms and becomes operational at birth, reaching adult activity levels by the age of five (3). Women who are depressed during pregnancy have been reported to have reduced vagus nerve activity, a finding also observed in their infants (3). These early influences on autonomic function may be linked with subsequent infant temperament and psychosocial development (4).

The vagus nerve was at the center of a key study in neuroscience history. The 1906 Nobel Prize for Physiology or Medicine went to Camillo Golgi and Santiago Ramón y Cajal, who bitterly disagreed about how nerve cells communicated: the former insisted that all nerve cells were connected through a single network, whereas Cajal believed that they were separate cells that needed some form of signaling to communicate with each other. The debate was resolved by Otto Loewi in 1921, who dreamt of an experiment that would once and for all resolve this issue, got up and scribbled a few notes so he could carry out the study in the morning; however, when he woke up, his writing was illegible. The next night, however, he had the same dream, and this time he ran to his laboratory, still in his pajamas. He placed a frog’s heart in a saltwater bath, then stimulated its vagus nerve to slow its pulse. He then poured the bathwater from this frog onto a second frog’s heart and found that the second heart now also beat slower, establishing that vagus nerve cells communicate by releasing a chemical (which he termed “vagusstoff,” now known to be acetylcholine) into the water. Loewi shared the 1936 Nobel Prize in Physiology or Medicine for this work.

## Reducing vagus nerve activity has therapeutic value in some clinical settings

Vagus nerve activity is essential for life, but too much of it can be harmful. A simple example is when a person faints: a distressing experience can provoke a large increase in vagus nerve activity, which decreases pulse and systemic blood pressure. In some cases, this drop in blood pressure can reduce cerebral perfusion pressure to the point of syncope. This can be triggered by a physical threat, such as a mouse running across one’s shoe, or by deeper psychological mechanisms. For example, Hero, in Shakespeare’s “Much Ado About Nothing,” faints at her wedding when she is unable to defend her virtue. It is unclear how losing consciousness is an adaptive response to either a scurrying mouse or an impugned reputation, which suggests that vagus nerve signaling can sometimes be harmful.

### Fear and bloating in the vagus

One medical condition in which reducing vagus nerve activity has been found to be beneficial is peptic ulcer disease. The vagus nerve contributes to gastric acid release, even if one is merely thinking about eating, as first noted by Ivan Pavlov (“psychic excitation of gastric secretion”) (5). Moreover, humans are one of the few species to continuously secrete stomach acid (along with beagles and trout). All that acid can contribute to peptic ulcer disease, which affects 1 in 10 U. S. citizens during their lifetime.

For a large part of the 20th century, a leading treatment for peptic ulcer disease was vagotomy, in which some or all of the vagus nerve fibers are surgically severed. This reduces acid secretion, thereby providing symptomatic relief. Studying the aftermath of this procedure also provides an opportunity to study the mind–body connection in relation to the pleasure of eating. Individuals who have undergone vagotomy report that they are less hungry, eat smaller meals, have reduced taste intensity, and are simply not as excited about food. Subsequently, in 2015, the FDA approved a vagus nerve blocking device to treat obesity in patients who have failed a supervised weight loss program.

### Circuits Circuits

The majority (80%) of vagus nerve fibers are afferent. Some evidence suggests that infections and poisons can take advantage of vagus afferents by using them as a conduit to transport toxic substances from the gut to the brain, but this remains an active area of investigation (6). For example, some experimental evidence supports that the influenza and SARS-CoV-2 viruses can invade and then ascend the vagus nerve in order to enter the brain (7).

The same mechanism may contribute to the development of Parkinson’s disease. The protein *α*-synuclein, which is critical to the pathogenesis of Parkinson’s disease, may initially form in the gut and then climb the vagus to enter the brain, as some experimental evidence supports the occurrence of retrograde axonal transport from the peripheral to the central nervous system, although other forms of vagus-mediated signaling between the enteric and central nervous systems may also contribute (8). Furthermore, some studies indicate that individuals who have undergone truncal vagotomy to alleviate peptic ulcer disease have a reduced risk of developing Parkinson’s disease (9). Together, these findings suggest the possibility that the vagus nerve may thus have great potential as a portal for therapies to access the brain.

## Increasing vagus nerve activity has therapeutic value in some clinical settings

### Seizure’s Palace

Vagus nerve stimulation (VNS) was first developed by a New York neurologist, James Corning, in the 1880s, in an attempt to control seizures. His methods were crude by today’s standards, and it took just over a century to develop a more refined approach.

The first modern device for VNS was implanted in 1988 by Penry and Dean (10) targeting seizure control. Clinical trials later established that VNS helps control seizures in certain patient subpopulations, and this intervention was approved by the FDA in 1997 as an adjunctive therapy for patients whose seizures are refractory to their medication (11). To date, more than 100,000 individuals with seizures have been treated with VNS.

Some patients using VNS to help control their seizures noticed that they also had reduced depression symptoms, consistent with studies documenting the capacity of vagus nerve signals to modulate mood. This finding was subsequently confirmed in clinical trials, and in 2005 the FDA approved VNS for the treatment of drug-resistant depression.

### Vagus golden heights

More recently, the same approach to VNS has been found to help restore movement in some patients whose arm was weakened by stroke. Pioneering preclinical work from the lab of Michael Kilgard demonstrated the functional benefits of providing VNS to animals with an experimental stroke (12). A company was established, with which I began consulting in 2011, to translate these favorable preclinical findings into human trials. I helped design two smaller studies, which produced favorable results (13, 14), and then a pivotal study which enrolled 108 subjects with arm weakness due to an ischemic stroke that had occurred at least 9 months prior. Half of the participants received VNS combined with rehabilitation therapy, while the other half received rehabilitation therapy only. Those in the VNS group were found to have significant improvement in arm movement status (15), and in 2021 the FDA approved VNS for this indication. The benefits of adding VNS were modest, however, fueling some controversy as to the clinical importance of the trial’s results (16). Recent findings suggest that VNS-related gains are sustained for at least 2 years (17), and real-world experience of using VNS in patients with hemiparetic stroke (in which this author was not involved) has been consistent with clinical trials (18), suggesting that some degree of clinical utility is likely present. Key challenges for future studies include understanding the mechanisms by which VNS may facilitate motor recovery after stroke; exploring whether other post-stroke complications can be improved by VNS, particularly post-stroke depression and seizures; and developing tools, such as those employing neuroimaging (19), that prospectively identify which patients are likely to respond to VNS combined with rehabilitation therapy. Other issues include determining whether benefits can be realized using non-invasive VNS approaches. With the growing uptake of this intervention, a robust health economic analysis will be important. While reports to date on the adverse event profile have been rather favorable, this issue also requires detailed assessment.

In light of these successes and given that the vagus nerve mediates a wide range of bodily functions from endocrine to immune, VNS is being studied as a treatment for many other conditions. For example, VNS can modulate immune system function and is therefore being evaluated for its ability to reduce the activity of autoimmune disorders such as rheumatoid arthritis and inflammatory bowel disease.

## Conclusion

The vagus nerve supports numerous bodily functions essential for life and is a key structure mediating the mind–body connection. Some conditions are helped by reducing vagus nerve signaling, while others are helped by increasing it. Improvements in technology may one day provide the tools for a personalized medicine approach that precisely controls vagus nerve activity, possibly by clinicians, therapists, and patients using a controller device. One might speculate that one day vagus nerve signaling may be modulated using a closed-loop system that adjusts activity in that nerve according to blood chemokine levels, signals in the gastrointestinal tract, or measures of activity elsewhere in the nervous system. This would be aided by the capacity to directly measure activity in an individual’s vagus nerves.

In “Do Androids Dream of Electric Sheep?,” Philip K. Dick describes the Penfield mood organ, which allows one to wake up and dial in their preferred emotional state for the day. That day may be a reality sooner than we think. In the future, one can imagine the invention of a vagus nerve device that is able to sense abnormal signaling in a patient such as the one described in the opening paragraph, prior to a high-valence anniversary (such as this patient’s 80th birthday), and then adjust the nerve’s activity to prevent his death.

## Data Availability

The original contributions presented in the study are included in the article/supplementary material, further inquiries can be directed to the corresponding author.
